# Reply to Hemmige and David

**DOI:** 10.1093/cid/ciz348

**Published:** 2019-05-02

**Authors:** Eili Y Klein, Katie K Tseng, Oliver Gatalo, Sara E Cosgrove

**Affiliations:** 1 Department of Emergency Medicine, Johns Hopkins School of Medicine, Baltimore, Maryland; 2 Department of Epidemiology, Johns Hopkins Bloomberg School of Public Health, Baltimore, Maryland; 3 Center for Disease Dynamics, Economics & Policy, Washington, DC; 4 Division of Infectious Diseases, Department of Medicine, Johns Hopkins University School of Medicine, Baltimore, Maryland

To the Editor—Methicillin-resistant *Staphylococcus aureus* (MRSA) remains among the leading causes of mortality in the United States due to antibiotic-resistant infections [1]. However, as we recently reported, rates of methicillin-susceptible *S. aureus* (MSSA) increased between 2010 and 2014 [2], as did the costs for treating these infections [3]. In fact, our estimates for 2014 found that the average costs of MSSA pneumonia and other infections (which are primarily skin and soft tissue infections) were higher than comparable MRSA infections [3]. These results utilized propensity score matching (PSM) to reduce biases and dependence on model formulation in the results.

Hemmige and David [4] expressed concern that the inclusion of patient length of stay (LOS) and the number of procedures performed in the analysis may have biased the outcomes by being one of the causal factors driving the differences in costs between MRSA and MSSA infections. In developing the paper, we included LOS as a matching parameter because there is also a causal relationship between LOS and the acquisition of hospital-acquired infections (HAIs) [5–7], and *S. aureus* is a common HAI-causing pathogen [1]. Additionally, a multitude of factors, not just infections, can affect a patient’s LOS, and we did not have information on infection timing. We were thus more concerned about the potential of matching patients with short and long LOSs that were due to other factors. We accounted for this in two ways. First, we matched on stratified LOS: ≤7, 8–14, 15–20, and 21+ days. Second, we conducted a subanalysis of patients with relatively short LOSs (≤10 days) and no mortality to reduce the bias from other factors driving LOS [3]. With regards to procedures, we included them in the match, as *S. aureus* infections are more likely to be attributed to invasive procedures than they are to cause additional procedures [5–7].

To assess the implications of these decisions, we reanalyzed the data for 2014, excluding LOS and procedures from the matching process. In addition, we included data for 2015 and 2016 to assess trends since 2014. We found that the results from the original paper [3], that MSSA infections might be more costly in 2014, continued in 2015 and 2016 (Figure 1A). Removing LOS and procedures from the PSM algorithm resulted in an increase in the magnitude of this difference for pneumonia and other infections, though septicemia remained unchanged (Figure 1B). Restricting the analysis to patients who were discharged alive with an LOS ≤ 10 days found the results of including LOS and procedures in matching (Figure 1C) were similar to the results when excluding LOS and procedures (Figure 1D). The impact of MRSA infections on LOSs has been estimated to be between 2 to 8 excess days of hospitalization, depending on the type of infection [8, 9]; thus, there is likely a causal relationship, as suggested by Hemmige and David [4]. However, not accounting for the endogeneity of infection risks related to longer lengths of stay, as we did when we stratified LOS, likely biases the results. Our findings point out the importance of taking into account the potential causal pathways in defining covariates for matching, but also highlight the difficulties in defining causal pathways in complicated hospital stays. These results also highlight the trade-off in using big data sets in health care, which are more generalizable but may not be able to account for some granular aspects of patient care. Nevertheless, the larger implication of our study, specifically the relative costliness of MSSA infections, remains true at a national level, regardless of methodology.

**Figure 1.**
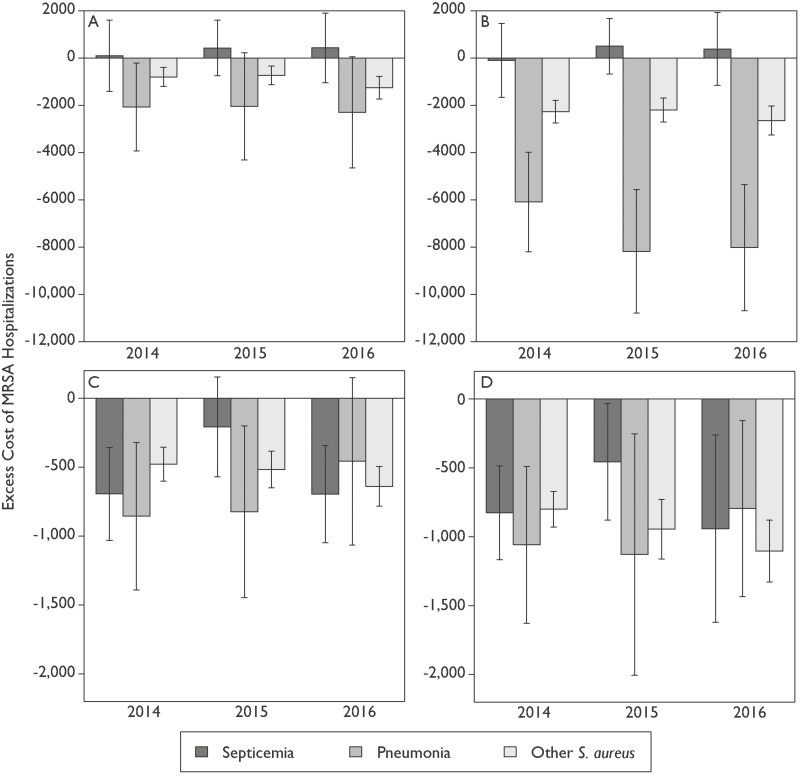
Comparison of different propensity score analyses of the excess cost of MRSA compared to MSSA hospitalizations by infection type, 2014–2016. The excess cost of MRSA-related hospitalizations, compared to MSSA-related hospitalizations, was measured as the mean cost of MRSA-related hospitalizations minus the mean cost of MSSA-related hospitalizations. The error bars are the 95% confidence intervals of the difference in the means, and negative values indicate that MRSA-related hospitalizations were, on average, less costly than similar MSSA-related hospitalizations. *A*, Estimated costs using a PSM algorithm accounting for LOS and numbers of procedures for 2014 (same as in the original paper) through 2016. *B*, Estimated cost without LOS and numbers of procedures in a PSM algorithm. *C*, Estimated costs for patients that were discharged alive with an LOS ≤ 10 days using a PSM algorithm including LOS and procedures for 2014 (same as in the original paper) through 2016. *D*, Estimated costs without LOS and number of procedures in a PSM algorithm for patients with an LOS ≤ 10. LOS was stratified as 0–7, 8–14, 15–20, and 21+ days to account for the endogeneity of infection risk in longer lengths of stay. Abbreviations: LOS, length of stay; MRSA, methicillin-resistant *Staphylococcus aureus*; MSSA, methicillin-susceptible *Staphylococcus aureus*; PSM, propensity score matching; *S. aureus*, *Staphylococcus aureus*.

## Notes

***Financial support.*** This work was supported by the Bill & Melinda Gates Foundation (grant number OPP1112355).

***Potential conflicts of interest.*** S. E. C. reports personal fees from Theravance and Novartis outside the submitted work. All other authors report no potential conflicts. All authors have submitted the ICMJE Form for Disclosure of Potential Conflicts of Interest. Conflicts that the editors consider relevant to the content of the manuscript have been disclosed.
